# Simultaneous Production of Lipids and Carotenoids by the Red Yeast *Rhodotorula* from Waste Glycerol Fraction and Potato Wastewater

**DOI:** 10.1007/s12010-019-03023-z

**Published:** 2019-05-10

**Authors:** Anna M. Kot, Stanisław Błażejak, Marek Kieliszek, Iwona Gientka, Joanna Bryś

**Affiliations:** 1 Department of Biotechnology, Microbiology and Food Evaluation, Faculty of Food Sciences, Warsaw University of Life Sciences, ul. Nowoursynowska 159C, 02-776 Warszawa, Poland; 2 Department of Chemistry, Faculty of Food Sciences, Warsaw University of Life Sciences, ul. Nowoursynowska 159C, 02-776 Warszawa, Poland

**Keywords:** Single-cell oil, Microbial carotenoids, Red yeast, Industrial wastes

## Abstract

Abstract

The objective of this study was to determine the possibility of simultaneous biosynthesis of lipids and carotenoids by the *Rhodotorula* yeast strains in media with waste glycerol and deproteinized potato wastewater and to determine the level of pollution reduction by media. On the basis of results obtained during the yeast microcultures in the Bioscreen C system, it was found that potato wastewater and glycerol can be used as components of media for *Rhodotorula glutinis*, *Rhodotorula mucilaginosa*, and *Rhodotorula gracilis* yeast strains. The amount of glycerol added to media higher than 10% significantly decreased the growth rate of yeast. The results of yeast culture in the laboratory shaker flasks showed a possibility of simultaneous production of lipids and carotenoids by *R*. *glutinis*, *R*. *mucilaginosa*, and *R*. *gracilis* yeast strains during cultivation in media containing only waste glycerol and deproteinized potato wastewater. A higher intracellular lipid content (approximately 15 g/100 g_d.w._) was obtained for *R*. *mucilaginosa* and *R*. *gracilis* yeast biomass after cultivation in experimental media with waste glycerol and potato wastewater. In conclusion, the yeast grown in media with potato wastewater supplemented with 3% or 5% glycerol synthesized carotenoids, and their content in biomass did not exceed 230 μg/g_d.w._

## Introduction

The ability of yeast to synthesize large amounts of lipids became known to scientists at the beginning of the twentieth century. Microorganisms that produce more than 20% lipids in a dry cellular substance are called oleaginous microorganisms [1]. At present, intensive research is being conducted worldwide on the possibility of producing microbial-derived lipids on an industrial scale. This stems primarily from the fact that this process is independent of climatic conditions, requires less manual intervention, has a short production cycle, and enables the fatty acid composition to be modified by regulating culture parameters. Yeasts are excellent producers of microbial lipids because of their high content in cellular biomass. These microorganisms exhibit a rapid growth rate and low nutritional requirements, and the fatty acid composition can be modified by changing the culture conditions [2]. The group of oleaginous yeasts includes yeasts of genus *Rhodotorula*, which apart from lipids can also synthesize other valuable cellular components such as carotenoids [3].

Carotenoids are industrially obtained by extraction from plant materials, chemical synthesis, and biosynthesis by microorganisms. Because of the constantly growing demand, new methods and sources of obtaining these compounds are sought. *Rhodotorula* yeast is capable of synthesizing three types of carotenoids: β-carotene, torulene, and torularhodin. β-carotene exhibits antioxidant and provitamin A properties. In the food industry, it is mainly used as a dye and as an ingredient in dietary supplements and sun tanning creams in the pharmaceutical and cosmetic industries [3]. Torulene and torularhodin are not found in food, and their impact on the human body has not yet been studied. These compounds also exhibit strong antioxidant properties, because of which they can be used in the future in the production of food, animal feed, and cosmetics [4].

At present, the high costs of microbial biosynthesis of lipids and carotenoids by yeast limit the possibility of using these processes on an industrial scale. One of the solutions to this issue may be the use of industrial waste as components of culture media [5]. The media used for the cultivation of yeast should primarily include assimilable sources of carbon, nitrogen, minerals, sulfur, and phosphorus compounds [6]. The waste that can be a valuable source of nitrogen compounds and minerals is deproteinized potato wastewater, which is produced during the production of potato starch. In the 2016/2017 season, approximately 200,000 tons of potato starch were produced in Poland [7]. The processing of one ton of potatoes produced approximately 7 m^3^ of potato wastewater [8].

This waste contains 2.9–4.3% of dry matter on average, and the dry matter consists of protein (0.93–1.57%), sugars (0.5–0.8%), fat (emulsified with water, 0.2%), and approximately 1% of mineral compounds. Hence, the values of the chemical oxygen demand index are generally higher than 20,000 mg/L. Because of the low content of reducing sugars, potato wastewater can be enriched with an additional source of carbon and energy in order to obtain a high biomass yield [9]. One such source is glycerol fraction, which is obtained from biodiesel production. It is estimated that approximately 10 kg of glycerol fraction is formed in the production of 100 kg of esters [10]. Glycerol fraction is the main by-product of the process and contains 40–70% glycerol, methanol residues (up to 14%), free fatty acids (up to 15%), water (2–3%), catalyst residues, soaps, and micronutrients such as sodium, magnesium, potassium, and calcium, It depends on the production technology, the raw material used, and the degree of methanol and catalyst recovery [10–12]. The combination of deproteinized potato wastewater and glycerol fraction allows the formation of a complex microbiological medium, which is a source of macro- and micronutrients required for the growth of the yeast [5].

The aim of the study was to determine the possibility of simultaneous production of lipids and carotenoids by selected yeasts of the genus *Rhodotorula* in media containing waste glycerol fraction as a source of carbon and deproteinized potato wastewater as a source of nitrogen and minerals. In the present study, the level of pollution reduction by media was also evaluated.

## Materials and Methods

### Biological Material

The biological materials of this study were three yeast strains of the genus *Rhodotorula*. *Rhodotorula glutinis* LOCKR13 was obtained from the Collection of Pure Cultures of the Lodz University of Technology (Poland), while the strains *R*. *mucilaginosa* ATCC 66034 and *R*. *gracilis* ATCC 10788 were obtained from the American Collection of Pure Cultures (USA). These microorganisms were stored on yeast extract peptone dextrose (YPD) medium slants at 4 °C.

### Waste Glycerol Fraction

In this study, the glycerol fraction was used, which was collected during the industrial production of biodiesel from rapeseed oil (Bioagra-Oil SA, Tychy, Poland).

The content of glycerol was determined by a chemical method proposed by Milchert et al. [13], involving oxidizing activity of meta-periodic acid to hydroxyl groups in glycerol. The results were given in grams per 100 g of waste glycerol fraction.

The content of methanol was determined by GC-MS (Shimadzu, GCMS-QP2010S) at HP-INNOWax capillary column (30 m × 0.25 mm × 0.25 μm) in Industrial Chemistry Research Institute in Warsaw. The results were given in grams per 100 g of waste glycerol fraction. The oven temperature was set at 35 °C (5 min); the temperature increase rate was 10 up to 220 °C (17 min). Helium was the carrier gas. The temperatures of the ion source and quadrupole were set at 200 °C and 150 °C, respectively. Identification of methanol was performed on the basis of Wiley Registry of Mass Spectral Database (9th edition).

The selected minerals were determined by inductively coupled plasma atomic emission spectroscopy (ICP-AES) (Thermo iCAP 6500) in the Analytical Center of WULS, Warsaw. For this, 2 mL of samples were mineralized (Buchi Digestion Unit K-435) using a mixture of nitric acid (5 mL) and perchloric acid (2 mL). Mixture after mineralization was transferred to 25 mL flasks by 5 mL of hydrochloric acid and deionizer water. Measurements were made at the appropriate wavelengths for each elements—Ca 315.8, 373.6, and 422.6 nm; K 766.4 and 769.8; Mg 279.5, 280.2, 285.2, and 382.9; Na 588.9 and 589.5; and P 177.4, 178.2, and 213.6. The results were given in grams per 100 mL of waste glycerol fraction.

### Deproteinized Potato Wastewater

Potato wastewater was prepared in laboratory conditions according to the methodology developed on the basis of the course of individual stages of potato starch production [14]. In deproteinized potato wastewater, the content of dry substance was determined by the gravimetric method (105 °C, SML 32/250, Zatmet). The total nitrogen and protein content was determined using the Kjeldahl method (Büchi Digestion Unit K-435, Büchi Distillation Unit K-355) with a conversion factor of 6.25 [15]. The content of reducing compounds, calculated per glucose, was determined spectrophotometrically at *λ* = 550 nm (UV1800 spectrophotometer, Rayleigh) by using 3,5-dinitrosalicylic acid [16]. The chemical oxygen demand index was determined by the dichromate method using Hach Lange cuvette tests (LCK014) in the Water Centre of the Warsaw University of Life Sciences. The content of calcium, potassium, magnesium, sodium, and phosphorus was determined by the ICP-AES method described above.

### Preparation of Inoculum

To prepare the inoculum, liquid YPD medium (20 g/L glucose, 20 g/L peptone, 10 g/L yeast extract, pH 5.0 ± 0.1) was used. This medium was inoculated with material collected from agar slants. Cultures were carried out in flasks containing 100 mL of the medium on a reciprocating shaker (SM-30 Control, Edmund Bühler), with a frequency of 200 rpm at 28 °C for 24 h.

### Culture Media

Two control media were used in the study: YPD medium and deproteinized potato wastewater. The experimental media were prepared from deproteinized potato wastewater and glycerol fraction, in which glycerol fraction was added in such an amount as to obtain an appropriate initial concentration of glycerol. To assess the growth of yeast during the cultures in the Bioscreen C system, experimental media supplemented with glycerol in doses of 30, 50, 100, 150, 200, and 250 g/L were used. On the basis of the growth results, doses of glycerol were chosen, and these doses were used for yeast cultures on a rotary shaker in flasks.

### Screening of the Growth by Using the Bioscreen C System

The culture of the *Rhodotorula* yeast in control and experimental media was performed using an automatic Bioscreen C system (Oy Growth Curves Ab Ltd., Finland). A total of 300 μL of the inoculated medium (1 × 10^6^ cells/mL) was added to one microwell of the microwell plate. The cultures were carried out for 120 h at 28 °C with intensive shaking. Optical density was measured at 2-h intervals by using a broadband filter (*λ* = 420–580 nm), as recommended by the equipment manufacturer. On the basis of the obtained results, the graphs of the optical density (OD) of culture from the time of cultivation were prepared, and the time of the adaptation phase (Δ*t*_lag_), log phase (Δ*t*_log_), and the minimum and maximum OD values in the logarithmic growth phase were determined. The coefficient of specific growth rate (*μ*_max_) was also calculated using the formula: *μ*_max_ = (lnOD_max_ − lnOD_min_)/Δ*t*_log_. The total increase in the OD of the culture (ΔOD) after 120 h was also determined.

### Experimental Culture Conditions

Yeast cultures were grown in 500 mL flasks on a reciprocating shaker at 200 rpm (SM-30 Control, Edmund Bühler) at 28 °C for 120 h. Media (90 mL) were inoculated with inoculum constituting 10% of the culture volume.

### Biomass Yield

The biomass yield was determined by the gravimetric method. For this purpose, 10 mL of the medium was centrifuged for 10 min at 6000×*g* (Centrifuge 5804R, Eppendorf), the supernatant was decanted, and the biomass was washed twice with deionized water. The wet cell biomass was dried at 105 °C (SML 32/250, Zatmet) to obtain a constant mass. The results were calculated in grams of dry weight per liter of culture medium (g_d.w._/L).

### Determination of Lipid Content in Yeast Biomass

The content of intracellular lipids was determined by the modified Bligh and Dyer method. The extraction of lipids was preceded by acid hydrolysis of the dry cellular matter. For this purpose, 10 mL of 1 M HCl was added to 200 mg of dry yeast biomass and incubated in a water bath at 60 °C (Memmert WNB14, Schwabach) for 2 h. The lipids were extracted with 20 mL of a mixture of chloroform and methanol (1:1). To separate these phases, 5 mL of 20% NaCl was added to the solution and centrifuged (3500×*g*/10 min, Centrifuge 5804R, Eppendorf). The lower phase was collected, and chloroform was evaporated under nitrogen atmosphere. The lipid content in the biomass of yeast was determined gravimetrically [17, 18] and expressed as grams per 100 g of dry weight.

### Analysis of the Composition of Fatty Acids

The fatty acids were esterified with a mixture of 2 M KOH in methanol. Methyl esters of fatty acids were analyzed in a gas chromatograph (TRACE™ 1300, Thermo Scientific) equipped with a flame ionization detector. The separation process was carried out on a capillary column RTX-2330 (60 m × 0.25 mm × 0.2 μm, Restek). The oven temperature was set at 50 °C (3 min); the temperature increase rate was 3 °C/min up to 250 °C (5 min). Nitrogen (1.6 mL/min) was the carrier gas. The temperatures of the injector and detector were set at 230 °C and 260 °C, respectively. The fatty acids were identified on the basis of standard retention time (Nu-Chek Prep, Inc., USA).

### Determination of Carotenoid Content in Yeast Biomass

The content of carotenoids in yeast biomass was determined by the spectrophotometric method [19]. Extraction of carotenoid pigments with a mixture of acetone (2 mL) and petroleum ether (2 mL) was preceded by disintegration of the yeast cell wall by 2 mL of DMSO and zirconium beads (*d* = 0.5 mm). The absorbance of the colored ether fraction was measured at *λ* = 457 nm (UV1800 spectrophotometer, Rayleigh), and the carotenoid content was calculated using the standard curve prepared for a β-carotene solution and expressed as microgram per gram of dry substance.

### Identification of Carotenoids

Carotenoids were identified by high-performance liquid chromatography coupled with a UV–Vis detector (Agilent 1200 Series, Palo Alto, CA, USA). The separation process of the mobile phase was carried out on a C18–2 analytical column (Bionacom, 250 mm × 4.6 mm, 5 μm) at a wavelength of 457 nm. The mobile phase was a mixture of acetonitrile, isopropanol, and ethyl acetate (4:4:2 *v*/*v*/*v*), and the flow rate was set at 0.7 mL/min (isocratically). The identification of β-carotene was based on the retention time of the standard (Sigma-Aldrich), while torulene and torularhodin were identified according to the retention times of the standards separated by thin-layer liquid chromatography (TLC) [14].

### Analysis of Postculture Media Composition

The content of glycerol, nitrogen, reducing sugars, and the COD index were determined in the postculture media according to the methods described above. On the basis of the obtained results, the content of glycerol, nitrogen, and reducing sugars and the decrease in the chemical oxygen demand index were calculated.

### Statistical Analysis

The results obtained from three independent experimental series were subjected to statistical analysis in the R program (version i386 2.15.3). The normal distribution of data was determined using the Shapiro–Wilk test, and the homogeneity of variance was determined using the Levene test. To determine the significance of differences between the average values, one-way analysis of variance (ANOVA) and Tukey’s test were performed. Analyses were conducted at the significance level *α* = 0.05.

## Results and Discussion

### Characteristics of Waste Glycerol Fraction and Deproteinized Potato Wastewater

The composition of glycerol fraction (Table 1) and deproteinized potato wastewater (Table 2) was to assess their suitability as the only components of culture media for yeast of the genus *Rhodotorula*. The glycerol fraction mainly consisted of glycerol (60.29%), which can be used by the *Rhodotorula* yeast as a source of carbon and energy [20]. According to the literature [21], the glycerol content in the glycerol fraction ranges from 40 to 70% and depends primarily on the production technology and the raw material used. For example, waste glycerol tested by Manowattan et al. [22] contained on average 56.3% glycerol, 6.1% water, 15.1% methanol, 10.8% lipid compounds, 6.1% ash, and 5.6% other pollutants. In the glycerol fraction used in this study, the methanol content was 3.30% and was a residue after the distillation process; thus, further attempts to recover would be unprofitable. In factories that are not equipped with a distillation station, the methanol content in the fraction may be as high as 14% [12]. Among the elements determined in the glycerin fraction, the highest proportion was sodium (1.1%), which was the result of using sodium hydroxide as a catalyst in the transesterification of fatty acids in the biodiesel production. Calcium, potassium, phosphorus, and magnesium were present in trace amounts (0.003–0.024%).

**Table 1:** Characterization of waste glycerol fraction (Bioagra-Oil S.A., Tychy)

Parameter	Unit	Average value ± SD
Glycerol	g/100 g	60.29 ± 0.38
Methanol	g/100 g	3.30 ± 0.10
Sodium	g/100 mL	1.107 ± 0.083
Calcium	g/100 mL	0.024 ± 0.003
Potassium	g/100 mL	0.007 ± 0.000
Phosphorus	g/100 mL	0.004 ± 0.001
Magnesium	g/100 mL	0.003 ± 0.001
pH	− log (H^+^)	12.1 ± 0.1

**Table 2:** Characterization of deproteinized potato wastewater

Parameter	Unit	Average value ± SD
Dry substance	g/100 mL	3.97 ± 0.05
Total nitrogen	g/100 mL	0.232 ± 0.007
Total protein	g/100 mL	1.45 ± 0.05
Directly reducing sugars	g/100 mL	0.73 ± 0.05
COD	mg O_2_/L	30,820 ± 527
Potassium	g/100 mL	0.431 ± 0.025
Phosphorus	g/100 mL	0.027 ± 0.002
Magnesium	g/100 mL	0.024 ± 0.001
Calcium	g/100 mL	0.016 ± 0.001
Sodium	g/100 mL	0.006 ± 0.000

The deproteinized potato wastewater was characterized by a high content of nutrients that are necessary for the proper growth of yeast. These included primarily nitrogen compounds (0.232 g/100 mL) necessary for microorganisms for the synthesis of amino acids and nucleic acids. There were also mineral components present in this waste, which determined the correct course of biochemical processes in cells. In addition, potato wastewater contained only a few simple sugars (0.73 g/100 mL), which are necessary to ensure favorable conditions for the growth and biosynthesis of lipids and carotenoids. The chemical oxygen demand index is one of the most important parameters that characterizes the degree of wastewater treatment. Lubiewski et al. [23] reported that for potato wastewater, the value of this indicator is above 20,000 mg /L. The value of COD ratio of potato wastewater used in this research was 30,820 mg/L, which confirmed the presence of a high content of organic and inorganic compounds in this waste. These values are too high to allow this waste to be disposed of in natural conditions [24].

### Growth Screening of Yeasts in the Control and Experimental Media

Microcultures of the *Rhodotorula* yeast were conducted in two control media—YPD and a medium containing deproteinized potato wastewater (PW), which was not enriched with an additional carbon source. A glycerol fraction collected from biodiesel production was added to the experimental media in such an amount that the final concentration of glycerol in the media ranged from 3 to 25%. The doses of glycerol to the experimental media were selected on the basis of a review of the literature, which shows that in order to obtain a high biomass yield and efficient biosynthesis of carotenoids and lipids, a high amount of carbon source compounds must be present in the culture medium. This is particularly important for the biosynthesis of intracellular lipids in yeast cells, and the initial molar ratio C/N plays a significant role [25, 26]. In the prepared experimental media, the molar ratio C/N ranged from 6/1 to 68/1 (Table 3).

**Table 3:** Molar C/N ration in control and experimental media

Culture medium	C/N ratio
YPD	1
PW	0.6
PW+G3%	6
PW+G5%	10
PW+G10%	22
PW+G15%	35
PW+G20%	51
PW+G25%	68

On the basis of results of microcultures from the Bioscreen C system (Table 4), it was found that the *Rhodotorula* yeast could grow in the control and experimental media. However, because of the different sensitivities of the tested yeast strains to high glycerol concentrations in the experimental media, the glycerol fraction must be added to the medium at the appropriate dose. The lowest resistance was found for the *R*. *gracilis* yeast strain, whose growth was completely inhibited in the medium with PW and 15% of glycerol. *Rhodotorula glutinis* yeast did not grow in a medium containing a 25% addition of glycerol, whereas the growth of *R*. *mucilaginosa* was inhibited in a medium containing 20% of this compound. Of the tested strains, only *R*. *glutinis* yeast showed greater or similar growth rate in media with potato wastewater and glycerol than in the control medium YPD (*μ*_max_ = 0.0356), and the highest value of this index (*μ*_max_ = 0.0398) was found in the PW+G3% medium. Furthermore, the addition of glycerol to PW reduced the growth rate of *R*. *mucilaginosa* and *R*. *gracilis*. The time of the adaptation phase was considerably lengthened, which may have resulted from the increase in the osmotic pressure of the media.

**Table 4:** Parameters characterizing the growth of studied *Rhodotorula* yeast strains in experimental media based on microculture in Bioscreen C system

Culture medium	*t*_lag_ [h]	*t*_log_ [h]	*μ*_max_ (h^−1^)
*Rhodotorula glutinis*			
YPD	2	18	0.0356
PW	2	18	0.0348
PW+G3%	2	18	0.0398
PW+G5%	2	18	0.0341
PW+G10%	8	20	0.0365
PW+G15%	18	20	0.0331
PW+G20%	18	22	0.0292
PW+G25%	No growth		
*Rhodotorula mucilaginosa*			
YPD	4	20	0.0322
PW	4	18	0.0318
PW+G3%	6	20	0.0287
PW+G5%	12	34	0.0210
PW+G10%	28	38	0.0176
PW+G15%	56	The logarithmic growth phase did not end up to 120 h	
PW+G20%	No growth		
PW+G25%	No growth		
*Rhodotorula gracilis*			
YPD	2	18	0.0401
PW	2	16	0.0353
PW+G3%	12	28	0.0230
PW+G5%	52	30	0.0213
PW+G10%	52	46	0.0134
PW+G15%	No growth		
PW+G20%	No growth		
PW+G25%	No growth		

Glycerol belongs to osmotically active substances and significantly affects the osmotic potential of the environment [27]. High concentrations of glycerol in the media caused the appearance of the so-called osmotic stress in the cells, which significantly decreased or inhibited the growth of yeasts. An increase in the osmotic pressure of the environment could also reduce the permeability of cell membranes and cause the closure of specific transport channels, because of which the yeast was unable to use the nutrients present in the medium [28]. Yeasts could also synthesized specific osmoregulation substances [29], including glutamate, γ-aminobutyrate, trehalose, proline, glycine betaine, or choline [30], which probably lengthened the adaptation phase. Another factor that limited the growth of yeast in experimental media was the presence of other impurities introduced with the crude glycerol fraction. The addition of glycerol to the culture medium increased the content of methanol and other compounds present in this waste, which in higher concentrations became toxic to yeast cells [31].

In the next part of the study, including yeast cultures in shaker flasks, for *R*. *glutinis* and *R*. *mucilaginosa* yeast strains, media with PW and 3%, 5%, and 10% doses of glycerol were selected. Because of the significantly slower growth rate of *R*. *gracilis* yeast in the medium with 10% of glycerol, this variant was excluded, and the cultures of this strain were carried out in media supplemented with 3% and 5% addition of this compound.

### Biomass Yield

**Fig. 1:**
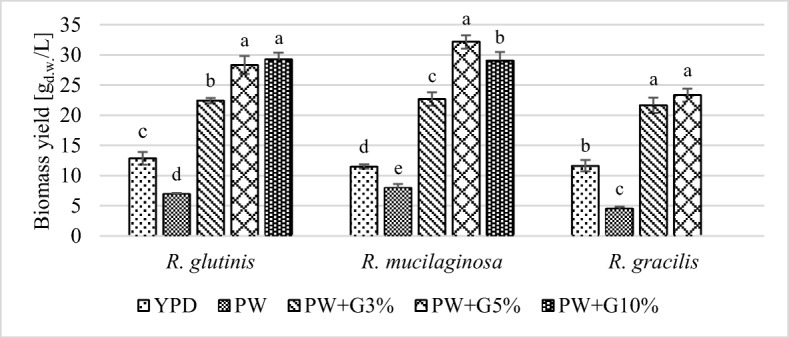
Biomass yield of *Rhodotorula* yeast after 120 h of cultivation in control and experimental media (a, b, c… indexes mean homogeneous groups, Tukey’s test, *α* = 0.05)

The tested yeast strains showed the ability to grow and biosynthesize lipids and carotenoids in media with PW and glycerol. To obtain high biomass yield, it was required to select the appropriate initial concentration of glycerol, depending on the yeast strain. After 120 h of cultures on the shaker, the highest biomass yield for *R*. *glutinis* and *R*. *mucilaginosa* was found in media with 5% and 10% glycerol addition (approximately 28–32 g_d.w._/L), while for *R*. *gracilis*, the highest biomass yield (approximately 20–22 g_d.w._/L) was obtained in media with 3% or 5% addition of this compound (Fig. 1). The determined values of cellular biomass in experimental media were significantly higher than those after cultivation in the control YPD medium.

Previous studies [20, 32, 33] have reported the use of glycerol as a carbon source for the cultivation of yeasts of the genus *Rhodotorula*. In these studies, various compounds (ammonium salts, yeast extract, or peptone) were used as a source of nitrogen. Saenge et al. [20] reported *R*. *glutinis* TISTR 5159 yeast culture in media containing 5% and 9.5% glycerol and ammonium sulfate as a source of nitrogen. The yield of cellular biomass was only 5.15–5.65 g_d.w._/L, while the yield obtained in the present study was approximately 18–21 g_d.w._/L. Karamerou et al. [33] used a yeast extract as a nitrogen source and glycerol as a carbon source at a dose of 3.5%. To obtain a high cell biomass yield (16.8 g_d.w._/L), cultures of *R*. *glutinis* CICC 31596 yeast were carried out for 192 h. The cellular biomass yields from the genus *Rhodotorula* obtained in the abovementioned studies were significantly lower than those achieved in this study. Therefore, PW can be considered a substitute for commonly used nitrogen source compounds such as ammonium sulfate or yeast extract.

### The Use of Components from the Medium by Yeast

The tested yeast strains of the genus *Rhodotorula* intensively metabolized reducing sugars, nitrogen compounds present in PW, and glycerol added into the media in the form of glycerol fraction. The total use of glycerol from culture media was dependent on the yeast strain and the level of glycerol added to the media. In a medium with 3% addition of glycerol, after 120 h, the total utilization of this compound was found to be above 97% (Table 5). After cultivation in the PW+G5% medium, the total glycerol utilization level varied from 63 to 92% depending on the strain. Further, the total glycerol utilization level after cultivation in PW+G10% medium for *R*. *glutinis* yeast and *R*. *mucilaginosa* was approximately 40%. To increase the use of glycerol in these media, the time of cultivation should be extended.

**Table 5:** The percentage level of glycerol, nitrogen, and reducing sugars usage from the media, finally media pH and level of the COD index reduction after 120 h of cultivation *Rhodotorula* yeast

Culture medium	Level of glycerol usage (%)	Level of nitrogen usage (%)	Level of reducing sugar usage (%)	Finally medium pH	Level of COD index reduction (%)
*Rhodotorula glutinis*					
YPD	–	32.1 ± 3.5	90.7 ± 0.1	8.7 ± 0.3	–
PW	–	24.5 ± 5.3	75.9 ± 3.4	9.3 ± 0.1	43.4 ± 5.3
PW+G3%	97.8 ± 1.1	62.1 ± 0.8	77.4 ± 6.3	8.7 ± 0.1	81.0 ± 2.6
PW+G5%	79.5 ± 3.6	61.1 ± 5.4	72.2 ± 1.8	8.7 ± 0.2	77.6 ± 3.5
W+G10%	39.5 ± 2.7	59.8 ± 7.0	72.7 ± 4.4	8.5 ± 0.2	49.1 ± 5.1
*Rhodotorula mucilaginosa*					
YPD	–	19.1 ± 4.6	91.5 ± 1.4	8.4 ± 0.2	–
PW	–	28.7 ± 6.7	71.6 ± 2.3	9.5 ± 0.1	49.0 ± 6.6
PW+G3%	98.0 ± 1.3	66.4 ± 2.2	72.6 ± 3.6	8.7 ± 0.2	83.2 ± 1.1
PW+G5%	91.9 ± 4.1	67.5 ± 3.3	73.9 ± 3.2	8.6 ± 0.2	82.6 ± 1.2
PW+G10%	41.1 ± 1.8	63.0 ± 6.1	74.9 ± 1.7	8.6 ± 0.1	54.7 ± 3.6
*Rhodotorula gracilis*					
YPD	–	33.6 ± 4.8	90.3 ± 1.8	8.5 ± 0.2	–
PW	–	32.1 ± 4.5	78.5 ± 2.1	9.4 ± 0.2	39.5 ± 6.6
PW+G3%	99.3 ± 0.2	65.0 ± 2.6	78.1 ± 2.4	8.4 ± 0.1	81.8 ± 1.3
PW+G5%	62.9 ± 2.7	52.5 ± 4.6	72.6 ± 3.5	8.4 ± 0.2	63.1 ± 3.6

The total level of nitrogen utilization from the experimental media by tested yeast strains of the genus *Rhodotorula* ranged from 52 to 67% (Table 5). The remainder was probably the nitrogen compounds in the form that were not assimilable by yeast. In addition to amino acids, peptides, and inorganic salts, PW contains Maillard reaction products, which are formed mainly in the deproteination stage. At a higher temperature, condensation occurs between the carbonyl groups of the sugars and the primary amino groups of the amino acids. The imines thus formed then undergo a spontaneous rearrangement to the so-called Amadori compounds, which in turn polymerize to brown products [34, 35]. Thus, the compounds formed as a result of the Maillard reaction were not used by yeasts during cultivation. The remaining nitrogen in the media could also be secondary yeast metabolites such as ammonia.

The content of reducing sugars in media decreased from approximately 0.7 to 0.2%. PW contains mainly simple sugars such as glucose, rhamnose, and galactose [23], of which the tested *Rhodotorula* yeast strains assimilate glucose, whereas the use of galactose and rhamnose is strain dependent [36]. Thus, it can be assumed that the residue in media with PW was galactose and rhamnose.

After 120 h of cultivation, the highest pH (9.3–9.6) was shown by control media. Strong alkalization also occurred in the experimental media, and the pH values (8.4–8.7) were similar for all tested yeast strains. Alkalization probably occurred due to secretion of secondary metabolites formed during the transformation of nitrogen compounds, which include ammonia. The reason for the alkalization could also be the degradation of AMP (adenosine monophosphate) to IMP (inosine monophosphate) and ammonium ions [37, 38]. It is considered that the optimal pH value of the cultivation medium for most species of the genus *Rhodotorula* ranges from 5.0 to 6.0 [39]; therefore, the alkalization of the medium may be a stressful factor for cells. It is known that alkalization primarily affects the metabolism and uptake of nutrients by yeast [40, 41]. However, in the present study, biomass yields and the level of nutrient utilization from the media were high, which indicates that the alkalization did not negatively affect the growth of the tested yeast strains.

The chemical oxygen demand is an important parameter that characterizes the degree of wastewater treatment. It is calculated as the amount of oxygen taken from the oxidant (potassium dichromate) for the oxidation of organic and some inorganic compounds such as nitrites, sulfides, and sulfites. The maximum value of COD for wastewater that can be discharged into the soil or waters according to the Regulation of the Polish Ministry of the Environment from 2014 is 125 mg/L. COD values were determined for deproteinized PW and experimental media before yeast culture and after 120 h. Accordingly, the reduction degree of COD for particular yeast cultures was calculated.

Cultures of yeast of the genus *Rhodotorula* in a medium containing PW reduced the COD index from 29,847 to 15,220–18,033 mg/L depending on the strain. The low total reduction rate (below 50%) in the control medium containing PW resulted from the weak use of nitrogen. Supplementation with glycerol from potato wastewater significantly increased the initial COD value of substrates to 70,997 (PW+G3%), 101,073 (PW+G5%), and 175,960 mg/L (PW+G10%). After 120 h of cultivation of the tested yeast strains of the genus *Rhodotorula*, the highest reduction rate (81.0–83.3%) and the lowest COD values (11,927–13,513 mg/L) were found in the medium with 3% glycerol addition. The values of COD in these conditions were significantly lower than those after cultivation in the control medium with PW, which was caused by the higher use of carbon and nitrogen sources.

### Biosynthesis of Lipids by the Yeast Strains and Fatty Acid Composition

**Fig. 2:**
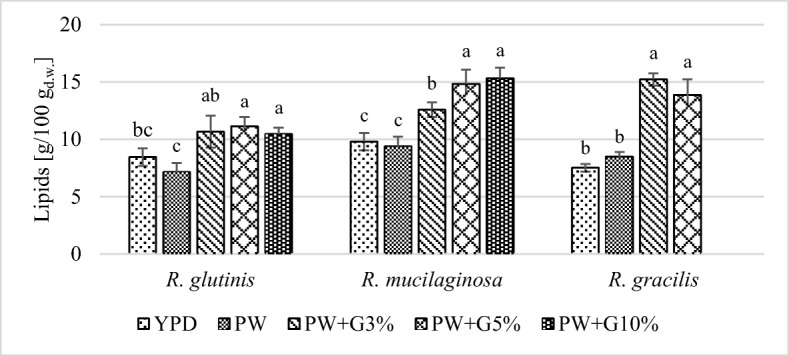
Total lipids content in *Rhodotorula* yeast biomass after 120 h of cultivation in control and experimental media (a, b, c… indexes mean homogeneous groups, Tukey’s test, *α* = 0.05)

The tested yeast strains synthesized intracellular lipids, and their content was dependent on the strain and the type of culture medium. After cultivation of the yeast strains in experimental media, the lipid content did not exceed 16 g/100 g_d.w._ (Fig. 2). It is worth noting that after the cultivation of *R*. *mucilaginosa* and *R*. *gracilis* yeast in media with PW and glycerol, the lipid content was significantly higher (approximately 15 g/100 g_d.w._) than in the control media (approximately 7–9 g/100 g_d.w._). This may have resulted from the fact that glycerol is the skeleton of the structure of triacylglycerol molecules, whereby its presence in the medium may induce the biosynthesis of these compounds by yeast [42].

In the experimental media used, the nitrogen content was probably too high for increased production of lipids in yeast cells. Under conditions of deficiency of nitrogen compounds in the culture environment, AMP deaminase is activated, which catalyzes the reaction of degradation of AMP to IMP and ammonium ions. Thereafter, citrate accumulates in the mitochondria, from where it is then transported to the cytoplasm and decomposed to acetyl-CoA and oxaloacetate by ATP citrate lyase. The acetyl-CoA molecules are a substrate for the synthesis of fatty acids [43]. In a culture medium containing a limited amount of nitrogen (yeast extract 0.5 g/L, ammonium sulfate 0.31 g/L) and glucose (50 g/L) as a carbon source, Jiru et al. [44] obtained biomass containing 56% lipids in the dry substance of the cell by using *Rhodotorula kratochvilovae* SY89 yeast.

The C/N molar ratio depends on the content of carbon and nitrogen source compounds in culture media. In the culture media with PW and glycerol *(3%, 5%, or 10%) used in this study, it ranged from 6/1 to 22/1 (Table 3). Low values of this parameter may be due to the fact that the yeast did not fit the criterion of oleaginous microorganisms. Papanikolaou and Aggelis [6] reported that a C/N ratio higher than 20 stimulates lipid biosynthesis by yeast, while lower values indicate a positive influence on the growth of cellular biomass, which was also observed in the present study. This is because the available forms of carbon and nitrogen are mainly used to meet the cells’ need related to multiplication and development. When the nitrogen availability in the medium is reduced, the rate of growth decreases and the excess carbon is used to synthesize lipids as a storage substance [45].

Another factor that could affect the low content of lipids in the yeast biomass was the high pH of culture media, which was above 8 (Table 5). The alkalization of the cultivation medium induces the expression of genes responsible for glucose metabolism, which results in the enhanced synthesis of polysaccharides such as trehalose [40, 41]. In the media containing both simple sugars and glycerol, there was an overproduction of intracellular polysaccharides instead of lipid biosynthesis. The profitability of microbial lipid production, in addition to the lipid content in biomass, is primarily determined by the yield of cellular biomass and the time of cultivation, which is expressed by indicators of volume efficiency and productivity of lipid biosynthesis (Table 6). The highest value of volume efficiency (4.46–4.76 g/L) and productivity (0.037–0.040 g/L/h) was obtained after 120 h of *R*. *mucilaginosa* culture in experimental media PW+G5% and PW+G10%. For *R*. *glutinis* yeast, the values of these indicators were significantly lower (3.1 g/L and 0.026 g/L/h, respectively), because of the lower intracellular lipid content in biomass. The volumetric yields after cultivation of *R*. *gracilis* yeast in the media PW+G3% and PW+G5% were similar and amounted to 3.2–3.3 g/L, which resulted from the lower yields of cellular biomass. The productivity of lipid biosynthesis in these conditions was 0.027 g/L/h. In the control media, the values of these indicators were several times lower than those in the experimental media (Table 6).

**Table 6:** The values of volumetric yield of lipid (g/L) and volumetric lipid productivity (g/L/h) in control and experimental media

Culture medium	*R*. *glutinis*		*R*. *mucilaginosa*		*R*. *gracilis*	
	*Y*_L_	*Q*_L_	*Y*_L_	*Q*_L_	*Y*_L_	*Q*_L_
YPD	1.08 ± 0.01	0.009 ± 0.000	1.13 ± 0.10	0.009 ± 0.001	0.88 ± 0.10	0.007 ± 0.001
PW	0.49 ± 0.04	0.004 ± 0.000	0.75 ± 0.12	0.006 ± 0.001	0.38 ± 0.02	0.003 ± 0.000
PW+G3%	2.39 ± 0.27	0.020 ± 0.002	2.86 ± 0.14	0.024 ± 0.001	3.30 ± 0.08	0.027 ± 0.001
PW+G5%	3.15 ± 0.07	0.026 ± 0.001	4.76 ± 0.25	0.040 ± 0.002	3.23 ± 0.19	0.027 ± 0.002
PW+G10%	3.06 ± 0.26	0.026 ± 0.002	4.46 ± 0.41	0.037 ± 0.003	–	–

*Y*_L_, volumetric yield of lipid; *Q*_L_,volumetric lipid productivity

Table 7 shows the percentages of palmitic, stearic, oleic, linoleic, linolenic, and arahinic acids. Microbial lipids also contained acids such as myristic, palmitoleic, heptadecanoic, behenic, and lignoceric acids, and their content did not exceed 2%. During cultivation in media with PW and glycerol, there was a reduction in the content of saturated fatty acids, i.e., palmitic and stearic acids, in lipids synthesized by the yeast strains tested compared with that in the control medium YPD. The content of monounsaturated acids increased due to the increase in oleic acid. The level of this acid was the highest (62–68%) for *R*. *mucilaginosa* and *R*. *gracilis* yeast strains, while for *R*. *glutinis* yeast, the level of this acid was significantly less (43–47%). In lipids synthesized by the yeast *R*. *glutinis* and *R*. *gracilis*, a significant amount of linoleic acid, whose share was > 17%, was also identified.

**Table 7:** The range of changes in the content of selected fatty acids (%) in lipids extracted from the *Rhodotorula* yeast biomass after 120 h of cultivation in experimental media (PW+G3%, PW+G5%, and PW+G10%) and in the control medium YPD

Fatty acid	*R*. *glutinis*		*R*. *mucilaginosa*		*R*. *gracilis*	
	Control medium (YPD)	Experimental media	Control medium (YPD)	Experimental media	Control medium (YPD)	Experimental media
C16:0	23.3	20.1–21.3	29.5	10.8–12.3	14.9	11.5–12.3
C18:0	15.6	7.9–9.4	22.7	9.3–10.9	3.2	3.1–4.9
C18:1	37.3	43.0–46.9	38.5	62.8–67.8	56.8	65.2–65.9
C18:2	11.4	14.5–17.3	1.6	1.9–3.0	15.9	10.6–12.6
C18:3	2.4	2.2–3.2	0.2	0.1–0.3	1.6	2.0–2.4
C20:0	2.8	1.9–2.5	1.5	1.7–3.0	2.9	1.1–1.4

*C16:0* palmitic acid, *C18:0* stearic acid, *C18:1* oleic acid, *C18:2* linoleic acid, *C18:3* linolenic acid, *C20:0* arachidic acid

Comparing the obtained results with the data on the composition of fatty acids of vegetable oils, it was found that the microbial lipids synthesized by *R*. *gracilis* yeast in media with PW and glycerol have a similar composition to olive oil. Orsavova et al. [46] determined the percentage of fatty acids in olive oil. The content of palmitic acid (16.5%), stearic acid (2.3%), oleic acid (66.4%), and linoleic acid (16.4%) was similar to that of acids in lipids synthesized by yeast *R*. *gracilis* in experimental media. Further, the overall share of SFAs (19.4%), MUFAs (71.1%), and PUFAs (18.2%) in olive oil was similar to that of the lipids synthesized by this yeast strain: SFAs (approximately 19%), MUFAs (approximately 67%), and PUFAs (approximately 14%) (Table 8).

**Table 8:** Percentages of fatty acids: saturated (SFAs), monounsaturated (MUFAs) and polyunsaturated (PUFAs) after 120 h of cultivation of *Rhodotorula* yeast in control and experimental media

Culture medium	SFAs	MUFAs	PUFAs
*Rhodotorula glutinis*			
YPD	45.6 ± 1.0	40.6 ± 2.6	13.8 ± 3.6
PW	35.4 ± 1.5	48.4 ± 3.1	16.2 ± 2.2
PW+G3%	34.4 ± 3.9	46.1 ± 4.8	19.5 ± 1.4
PW+G5%	33.9 ± 1.8	48.9 ± 1.4	17.2 ± 2.6
PW+G10%	35.4 ± 4.3	45.1 ± 2.5	19.5 ± 1.8
*Rhodotorula mucilaginosa*			
YPD	57.4 ± 3.6	40.0 ± 3.4	2.6 ± 0.4
PW	46.4 ± 4.2	49.6 ± 4.7	4.0 ± 0.5
PW+G3%	27.5 ± 0.3	69.4 ± 0.7	3.1 ± 0.6
PW+G5%	29.1 ± 0.3	68.5 ± 0.5	2.4 ± 0.4
PW+G10%	31.5 ± 3.5	64.7 ± 3.4	3.8 ± 0.1
*Rhodotorula gracilis*			
YPD	23.8 ± 0.8	58.3 ± 1.8	17.9 ± 2.1
PW	22.7 ± 3.0	63.7 ± 1.7	13.6 ± 1.5
PW+G3%	19.0 ± 4.2	68.0 ± 2.8	13.0 ± 2.3
PW+G5%	17.9 ± 1.8	67.0 ± 1.9	15.1 ± 1.9

### Biosynthesis of Carotenoids by the Yeast and Their Profile

**Fig. 3:**
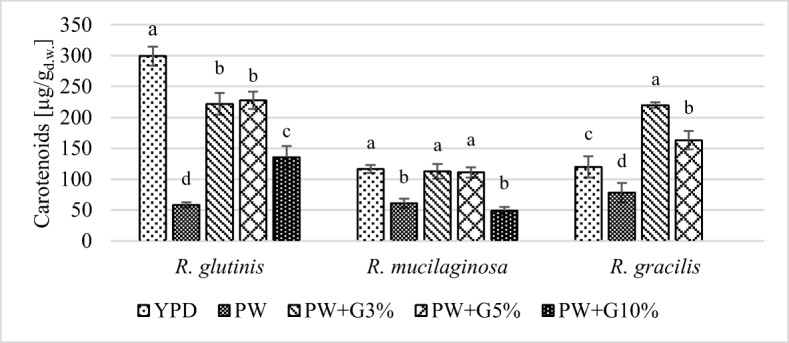
Total carotenoids content in *Rhodotorula* yeast biomass after 120 h of cultivation in control and experimental media (a, b, c… indexes mean homogeneous groups, Tukey’s test, *α* = 0.05)

The content of carotenoids in biomass depended on the yeast strain and the composition of the culture medium (Fig. 3). *Rhodotorula glutinis* synthesized significantly less total carotenoids (135–228 μg/g_d.w._) in the experimental media with PW and glycerol than in the YPD control medium (300 μg/g_d.w._). *Rhodotorula mucilaginosa* biomass was characterized by the lowest total carotenoid content among the tested strains. The highest total carotenoid content of these compounds (ok. 110 μg/g_d.w._) was found after cultivation in the YPD medium and in experimental media PW+G3% and PW+G5%. Similar to *R*. *glutinis* yeast, a decrease was noted in the total carotenoid content in *R*. *mucilaginosa* yeast biomass from the control medium PW and the experimental medium PW+G10%. *Rhodotorula gracilis* yeast synthesized significantly more total carotenoids when grown in a medium with PW and 3% glycerol (220 μg/g_d.w._) than in the control medium YPD (120 μg/g_d.w._). El-Banna et al. [47] proposed that the content of carotenoids in yeast biomass could be considered low for amount < 100 μg/g_d.w._, the average for 101–500 μg/g_d.w._, and high for > 500 μg/g_d.w._. On the basis of this criterion, it can be considered that the tested yeast strains of the genus *Rhodotorula* were average producers of carotenoids.

Analysis of the obtained results indicates that 10% concentration of glycerol inhibited the biosynthesis of carotenoid pigments by yeast. The phenomenon of limiting the production of carotenoids by *R*. *glutinis* yeast in media containing a high concentration of glycerol was reported by Saenge et al. [20] and Razavi et al. [48] for the yeast *Sporobolomyces ruberrimu*s. High glucose concentration in the medium can also lead to a reduction in the efficiency of carotenoid biosynthesis by the *Rhodotorula* yeast [49]. In the yeast *Phaffia rhodozyma* (synonym *Xanthophyllomyces dendrorhous*), increased glucose suppresses the biosynthesis of carotenoids due to the occurrence of the Crabtree effect [50, 51]. Although yeasts of the genus *Rhodotorula* are considered to be Crabtree-negative [36], some strains may produce ethanol [52]. Perhaps, the reduction of carotenoid biosynthesis noted in the present study in media with 10% added glycerol was due to the Crabtree effect.

The highest volumetric yield of carotenoid biosynthesis (6.46 mg/L) was found after the cultivation of *R. glutinis* yeast in the medium with PW supplemented with 5% glycerol. In these conditions, the productivity of carotenoid biosynthesis was 0.054 mg/L/h. For the remaining strains, *Y*_Car_ and *Q*_Car_ values did not exceed 5 mg/L and 0.042 mg/L/h, respectively (Table 9). Buzzini and Martini [53] cultivated *R*. *glutinis* DBVPG 3853 yeast in media containing various sources of carbon and nitrogen. The high content of total carotenoids (630 μg/g_d.w._) synthesized yeast cultivation in medium with concentrated grape must. However, due to the much lower biomass yield, the value of volumetric biosynthesis yield (5.95 mg/L) was comparable with that obtained in this study.

**Table 9:** The values of volumetric yield of carotenoids (mg/L) and volumetric carotenoids productivity (mg/L/h) in control and experimental media

Culture medium	*R*. *glutinis*		*R*. *mucilaginosa*		*R*. *gracilis*	
	*Y*_Car_	*Q*_Car_	*Y*_Car_	*Q*_Car_	*Y*_Car_	*Q*_Car_
YPD	3.84 ± 0.19	0.032 ± 0.002	1.34 ± 0.04	0.011 ± 0.000	1.40 ± 0.24	0.012 ± 0.002
PW	0.40 ± 0.04	0.003 ± 0.000	0.48 ± 0.05	0.004 ± 0.000	0.35 ± 0.04	0.003 ± 0.000
PW+G3%	4.98 ± 0.48	0.042 ± 0.004	2.56 ± 0.27	0.021 ± 0.002	4.76 ± 0.27	0.040 ± 0.002
PW+G5%	6.46 ± 0.53	0.054 ± 0.004	3.57 ± 0.38	0.030 ± 0.003	3.82 ± 0.52	0.032 ± 0.004
PW+G10%	3.96 ± 0.39	0.033 ± 0.003	1.42 ± 0.24	0.012 ± 0.002	–	–

*Y*_Car_,volumetric yield of carotenoids; *Q*_Car_, volumetric carotenoids productivity

**Fig. 4:**
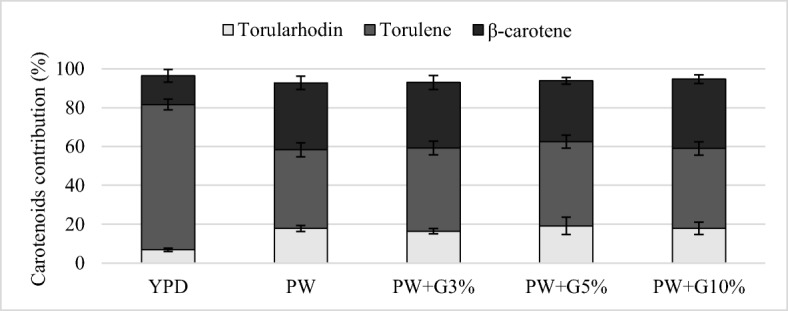
Carotenoid profile synthesized by *R*. *glutinis* after 120 h of cultivation in control and experimental media

**Fig. 5:**
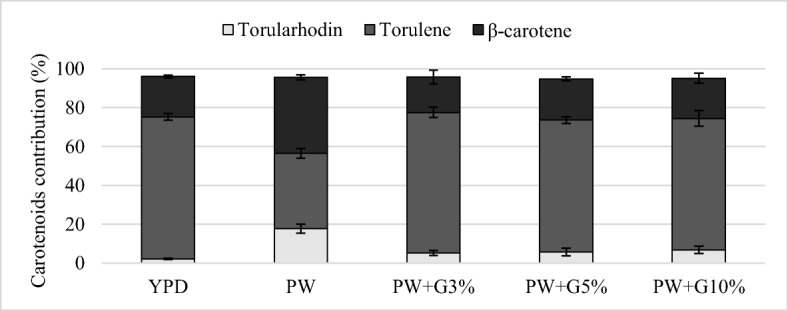
Carotenoid profile synthesized by *R*. *mucilaginosa* after 120 h of cultivation in control and experimental media

**Fig. 6:**
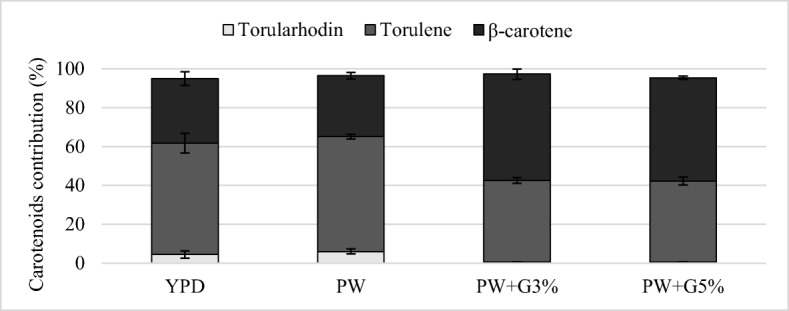
Carotenoid profile synthesized by *R*. *gracilis* after 120 h of cultivation in control and experimental media

The analysis of the profile showed different proportions of β-carotene, torulene, and torularhodin depending on the yeast strain and the composition of the medium (Figs. 4, 5, 6). During cultivation in the YPD control medium, *R*. *glutinis* yeast synthesized the largest amount of torulene (approximately 75%). The use of culture medium with PW and glycerol contributed to the increase in the proportion of β-carotene (31.4–36.5%) in comparison with the control culture. The addition of glycerol to PW contributed to the increase in β-carotene biosynthesis, and the proportion of this compound was approximately 54%. In these conditions, a lower proportion of torulene was noted (approximately 42%). The carotenoid fraction synthesized by *R*. *mucilaginosa* yeast was dominated by torulene, and its proportion after cultivation in the YPD medium and experimental media was similar and amounted to approximately 70%. The use of PW for the cultivation of *R*. *glutinis* and *R*. *mucilaginosa* yeast resulted in a significant increase in the production of torularhodin compared with that in the culture carried out in the control medium YPD. The highest proportion of this carotenoid fraction (17.9%) was found after the cultivation of *R*. *glutinis* yeast in the PW medium, and this strain synthesized the highest amount of torularhodin among the tested strains.

## Conclusions

The study showed that it is possible to simultaneously biosynthesize lipids and carotenoids by *R*. *glutinis*, *R*. *mucilaginosa*, and *R*. *gracilis* yeast when growing in a medium containing only waste glycerol and deproteinized PW. The use of these substrates resulted in increased lipid biosynthesis in the cells of yeast strains (10.5–15.2 g/100 g_d.w._) as compared with that in the YPD control medium (7.1–9.8 g/100 g_d.w._). The fatty acid composition of the lipid fractions was dependent on the yeast strain and culture conditions, with oleic, palmitic, stearic, and linoleic acids dominating in them. On the basis of the profile of individual fatty acids and the proportions of SFAs, MUFAs, and PUFAs, it was found that the lipids synthesized by *R*. *gracilis* yeast in a medium with PW and glycerol had a composition similar to that of olive oil. At the same time, the yeast synthesized carotenoid pigments with an average yield (approximately 230 μg/g_d.w._), and the analysis of the profile showed that the highest proportions were found for torulene and β-carotene. The results of the present research revealed that biomass of tested yeast strains obtained after cultivation in media with waste glycerol and deproteinized PW could become a source of microbial lipids containing natural antioxidants in the form of carotenoids. These compounds also exhibit the properties of provitamin A, which enables to use the yeast biomass, particularly that of *R*. *gracilis*, as a valuable feed additive.
